# Fish mapping of rdna and novel tandem repeats in *Citrus* species

**DOI:** 10.3897/compcytogen.20.190408

**Published:** 2026-06-30

**Authors:** Yuliya N. Smotrova, Mikhail Gr. Monakhos, Olga V. Razumova, Dmytry V. Romanov

**Affiliations:** 1 All-Russia Research Institute of Agricultural Biotechnology, Timiryazevskaya 42, 127550 Moscow, Russia Moscow Institute of Physics and Technology Dolgoprudny Russia; 2 Moscow Institute of Physics and Technology, Institutsky lane 9, 141700, Dolgoprudny, Moscow region, Russia Russian State Agrarian University – Moscow Timiryazev Agricultural Academy Moscow Russia; 3 Russian State Agrarian University – Moscow Timiryazev Agricultural Academy, Timiryazevskaya street 49, 127434 Moscow, Russia All-Russia Research Institute of Agricultural Biotechnology Moscow Russia

**Keywords:** *
Citrus
*, FISH, oligo probes, ribosomal DNA, species-specific repeats

## Abstract

Cytogenetic analysis of *Citrus* Linnaeus, 1753 species is complicated by the small size and morphological similarity of their chromosomes. In this study, we applied the fluorescence *in situ* hybridization (FISH) method using rDNA probes, as well as newly developed oligonucleotide probes based on previously uncharacterized tandem repeats identified in citrus genomes. Four tandem repeats (CL1, CL14, CL262, and CL122) were mapped to the chromosomes of five *Citrus* species: *Citrus medica* Linnaeus, 1753, *Citrus × aurantiifolia* (Christmann, 1777) Swingle, 1913, *Citrus × limon* (Linnaeus, 1753) Osbeck, 1765, *Citrus myrtifolia* Rafinesque, 1838, and *Citrus × latifolia* Tanaka, 1951. The repeats exhibited distinct and partially species-specific distribution patterns: CL1 was detected in all species, whereas CL262, CL122, and CL14 exhibited more restricted localization. A comparative analysis of their chromosomal distribution in relation to 45S- and 5S-rDNA loci revealed differences between the conserved regions of ribosomal DNA and the more dynamic tandem repeats. The identified repeats represent useful cytogenetic markers for chromosome identification, karyotyping, and comparative analysis within the genus *Citrus*.

## Introduction

The genus *Citrus* Linnaeus, 1753 (family Rutaceae) comprises evergreen trees and shrubs originating from a region including Assam (India), Myanmar, and southern China (Alexandrov and Romanov 2024). Citrus plants are widely cultivated throughout the world, particularly in tropical and subtropical regions (Nicolosi et al. 2000; Palangasinghe et al. 2024). Modern genomic and biochemical studies have revealed that cultivated citrus species are the result of interspecific hybridization among four principal taxa *Citrus reticulata* Blanco, 1837, *Citrus maxima* (Burman, 1755) Merrill, 1917, *Citrus medica* Linnaeus, 1753 and *Citrus micrantha* Wester, 1915 (Curk et al. 2016; Wu et al. 2018; Alexandrov and Romanov 2024).

The citrus genome is characterized by a diploid chromosome set (2n = 18), with the exception of some polyploid hybrids. The small size of the chromosomes (typically 2–4 μm) and the similar morphology among *Citrus* species make it difficult to identify chromosomes without specific markers (Lan et al. 2016; Deng et al. 2020; He et al. 2020).

Repetitive sequences constitute a significant portion of eukaryotic genomes (Mehrotra and Goyal 2014). In citrus fruits, repetitive sequences may be organized into tandem arrays of satellite DNA (satDNA), minisatellites (VNTRs), microsatellites (SSRs), or may represent mobile elements (retrotransposons, DNA transposons). For example, in the clementine genome (*Citrus × clementina* Tanaka, 1948), they account for just under half of the total DNA length (Mehrotra and Goyal 2014; Deng et al. 2019). Repetitive elements may play an important role in the evolution of *Citrus*, influencing changes in chromosomal structure, and phenotypic variation – for example, the activation of the Ruby gene and the accumulation of anthocyanins leading to the “blood-red” color of fruits, the emergence of apomixis, and changes in fruit acidity (Butelli et al. 2012, 2017; Giraud et al. 2025). Transposons constitute the majority of repeats in citrus, highlighting the dominant role of dispersed repeats in the process of speciation (Giraud et al. 2025). Mapping highly variable loci, such as species-specific satellite repeats and polymorphic rDNA clusters, allows the identification of subtle genomic differences between closely related species and hybrids (Xiao et al. 2025).

Understanding the distribution of DNA repeats is of great importance for studying genomic relationships and plant phylogeny. Conservative 5S and 45S rDNA sequences are easily detected in hybridization experiments due to their high copy number, making them classic cytogenetic markers (Barkov et al. 2007; Roa and Guerra 2012; Lan et al. 2016; Tourdot and Grob 2023). Identifying specific repetitive sequences for FISH markers typically requires a combination of bioinformatic analysis of genomic data and molecular biology methods. For this, in a study by Deng et al., the authors annotated satellite and centromeric repeats *de novo* using bioinformatic analysis of the clementine genome and successfully used them as chromosome-specific markers (Deng et al. 2019).

The citrus genome is characterized by high heterozygosity and admixture from ancestral species, which complicates the interpretation of repetitive sequences (Wu et al. 2018). In citrus, frequent instances of hybridization are accompanied by changes in copy number and rDNA localization, which correlates with stress adaptation (Deng et al. 2019; Goffová and Fajkus 2021). The 45S rDNA sequence is more conserved than the 5S sequence, allowing the use of universal probes for hybridization. However, the chromosomal positions of these regions are subject to evolutionary changes, and even with the same number of loci, their size and FISH signal intensity may vary (Roa and Guerra 2012). For example, the citrus-related genus *Poncirus* Rafinesque, 1938 possesses a site with a linked 5S-45S locus, which is not observed in pure citrus species and reliably identifies *Poncirus × citrus* hybrids (Brasileiro-Vidal et al. 2007). Several studies have emphasized that 5S and 45S rDNA serve as valuable cytogenetic markers due to their conserved nature. For example, in a study by Deng et al., a set of probes including 5S and 45S rDNA was developed to identify all chromosomes of clementine and related citrus species (Deng et al. 2019; Deng et al. 2020). Early studies have shown that most citrus species have between two and six 45S rDNA loci per karyotype, while the number of 5S rDNA loci is usually two, with a few exceptions (Deng et al. 2019).

The aim of this study was to map new repetitive sequences selected from the *Citrus* repeatome in the chromosomes of five *Citrus* species to evaluate their potential as cytogenetic markers. New tandem repeats and rDNA probes will allow the identification of both conserved and divergent features of genomic architecture for the comparison of closely related species.

## Material and methods

### Plant material

We took plants from the All-Russia Research Institute of Agricultural Biotechnology collection: *Citrus × latifolia* Tanaka, 1951, *Citrus medica* cv Uraltau, *Citrus myrtifolia* Rafinesque, 1838 (*Citrus × aurantium* Linnaeus, 1753 in the Swingle classification), *Citrus × limon* (Linnaeus, 1753) Osbeck 1765 cv Novogruzinsky, *Citrus × aurantiifolia* (Christmann, 1777) Swingle 1913 (Suppl. material 1).

### Chromosome preparation

For mitotic analysis, we pretreated root meristems from adult plants *C. × aurantiifolia*, *C. medica*, *C. × latifolia*, *C. × limon* and *C. myrtifolia* with 1-Bromonaphthalene for 18–20 h at 4 °C and then fixed them in ethanol:acetic acid (3:1, v/v) for 24 h at -20 °C. We prepared chromosomal preparation according to the protocol published in Romanov et al. (2020).

### DNA probes

For fluorescence *in situ* hybridization (FISH), we used universal 45S and 5S rDNA probes and specific oligonucleotide tags.

To develop the oligo-probes, we employed satellite repeat and LTR element sequences that we had annotated using RepeatExplorer2 (Novák et al. 2013; Monakhos et al. 2025). We designed specific oligonucleotide labels for hybridization based on the selected sequences using the Primer-BLAST tool (Ye et al. 2012). The fluorophore-labeled oligonucleotides were synthesized by SINTOL LLC (Moscow, Russia). Table 1 provides detailed information on the resulting labels (Table 1).

**Table 1.** Sequence table of DNA probes.

**Probe Name**	**5'-3' sequence**	**Dye**
CL14	CATAGAGGGCGTTCAAGCCGTTTCTGGACGTAAAACCAG	ROX
CL122	TTGGTGCATTGGAAGAGGGCCTCATCTCCAGTTGA	ROX
CL262	TTGTGCACTACTTCCTCCTGTCTCCATTGCTTTTCACTTGCTTCA	ROX
CL1	AAGTTCGTCCAGCGGAAAAATGCCCAAAAAACGGGTGGGCTATA	FAM
5S1	GGGTGCGATCATACCAGCACTAATGCACCGGATCCCATCAG	FAM
5S2	AACTCCGAAGTTAAACGTGCTTGGGCGAGAGTAGTACTAAG	FAM
5S3	ATGGGTGACCTCTTGGGAAGTCCTCGTGTTGCACCCCC	FAM
18S1	CTGGTTGATCCTGCCAGTAGTCATATGCTTGTCTCAAA	ROX
18S2	TCCAAGGAAGGCAGCAGGCGCGCAAATTACCCAAT	ROX
18S3	GTAACAAGGTTTCCGTAGGTGAACCTGCGGAAG	ROX

### Fluorescent *in situ* hybridization

We performed the FISH procedure according to Kuznetsova et al. (Kuznetsova et al. 2019). We followed the described protocol for the initial steps, up through the post-hybridization wash on the second day. We then washed the slides three times in 0,2 × SSC solution at 42 °C for a total of 10 minutes (1:3:6 min). This was followed by three 5-minute washes in TNT buffer (0,05% Tween 20). A final rinse was carried out in 2 × SSC for 5 min. Subsequently, the slides were stained with DAPI (4',6-diamidino-2-phenylindole) (1:100 in 2 × SSC) for 10 min after washing, then slides were mounted in Vectashield medium (Vector Laboratories, United States) and analyzed on Leica DM6 B epifluorescence microscope. Selected images were captured with DFC 9000 GTC (Leica). We analyzed 25–30 metaphase plates in each species with DRAWID and visualized with Photoshop to place probes on ideograms.

### Supporting tools

We used the DeepSeek AI model (DeepSeek R1) to ensure linguistic accuracy and compliance with the academic style of the article. The model was asked to translate the original Russian text using a specially designed set of instructions, paying special attention to the passive voice, technical accuracy and conciseness. The questions were as follows: 1. Criticize the revised statement as a reviewer, pointing out any shortcomings or areas requiring further improvement. *Initial draft* 2. Translate the following Russian text into formal, academic English of a scientific paper. Use active voice where appropriate, maintain precise scientific terminology, and ensure the text is concise and objective.

## Results

In this study, several previously uncharacterized tandem repeats (CL1, CL14, CL262 and CL122) were identified and localized on the chromosomes of five *Citrus* species using fluorescence *in situ* hybridization. In addition, the chromosomal positions of the classical cytogenetic markers 45S and 5S rDNA were determined (Suppl. material 1 fig. 6S-10S). Chromosome pairs were assigned based on chromosome morphology in DAPI-stained metaphases and the combined pattern of FISH signals on well-spread metaphase plates, and ideograms were constructed from the most representative plates (Suppl. material 2: figs S1–S5).

### Repeat DNA Localization

**Figure 1.**
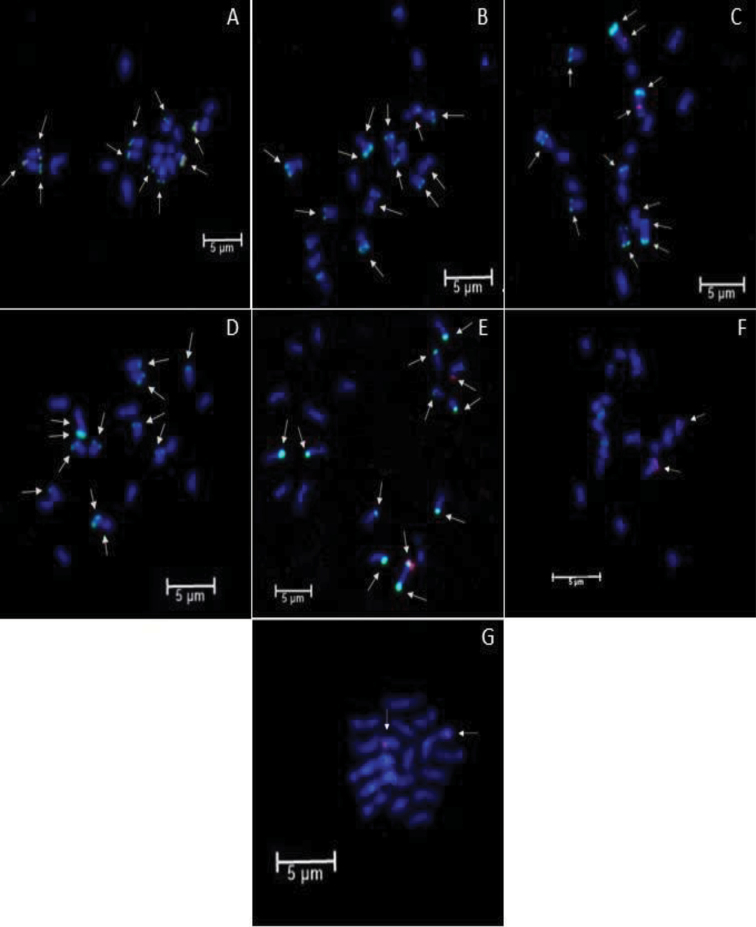
Chromosomal localization of probes. **A***C. × limon*: CL1 (green) and CL14 (red) **B***C. medica*: CL1 (green) and CL14 (red) **C***C. × latifolia*: CL1 (green) and CL262 (red) **D***C. × aurantiifolia*: CL1 (green) and CL262 (red) **E***C. myrtifolia*: CL1 (green) and CL122 (red) **F***C. × latifolia*: CL14 (red) **G***C. × aurantiifolia*: CL14 (red). White arrows indicate hybridisation sites. Scale bar: 5 µm.

The satellite repeat CL1 was localized in the subtelomeric regions of chromosomes of all studied species (Fig. 1). The number and intensity of the hybridization signals varied between species. *C. medica* exhibited signals on six chromosome pairs, *C. × latifolia* on four pairs, and *C. × aurantiifolia* on five pairs. In *C. × limon*, signals were localized to four chromosome pairs and an additional signal was detected on chromosome 9, while *C. myrtifolia* displayed signals on five chromosome pairs.

The CL14 repeat was identified and localized in the studied species. CL14 signals were detected on chromosome pairs 1 and 3 in *C. medica* in proximal regions (Fig. 1B). In *C. × limon*, signals were found on chromosome pairs 2 and 4 in subtelomeric regions (Fig. 1A), in *C. × latifolia* on chromosome pair 4 in a subtelomeric region (Fig. 1F) and in *C. × aurantiifolia* on chromosome pair 3 (Fig. 1G). No CL14 signals were detected in *C. myrtifolia*. A close co-localization of the CL14 and CL1 repeats was observed in *C. × limon* on chromosome pairs 2 and 4.

Hybridization signals of the CL262 repeat were observed in *C. × aurantiifolia* and *C. × latifolia*. In *C. × latifolia*, the signals were located in the pericentromeric region of chromosome pair 1 and in the subtelomeric region of chromosome 5 (Fig. 1C). In *C. × aurantiifolia*, signals were also present in the pericentromeric region of chromosome pair 1 and in the subtelomeric region of chromosome 4 (Fig. 1D). No hybridization signals were detected in the other species examined.

The CL122 repeat was localized to the subtelomeric regions of chromosomes 2 and 4 in *C. myrtifolia* (Fig. 1E). No hybridization signals were detected in the other species. On chromosome 2, it was found to be co-localized with the satellite repeat CL1.

### 5S and 45S rDNA localization

5S rDNA loci were detected on chromosomes 4 and 6 in *C. × aurantiifolia*, on chromosomes 6 and 9 in *C. medica*, on chromosome pair 9 in *C. × latifolia*, on chromosome 6 in *C. × limon*, and finally, on chromosomes 7 and 9 in *C. myrtifolia*.

**Figure 2.**
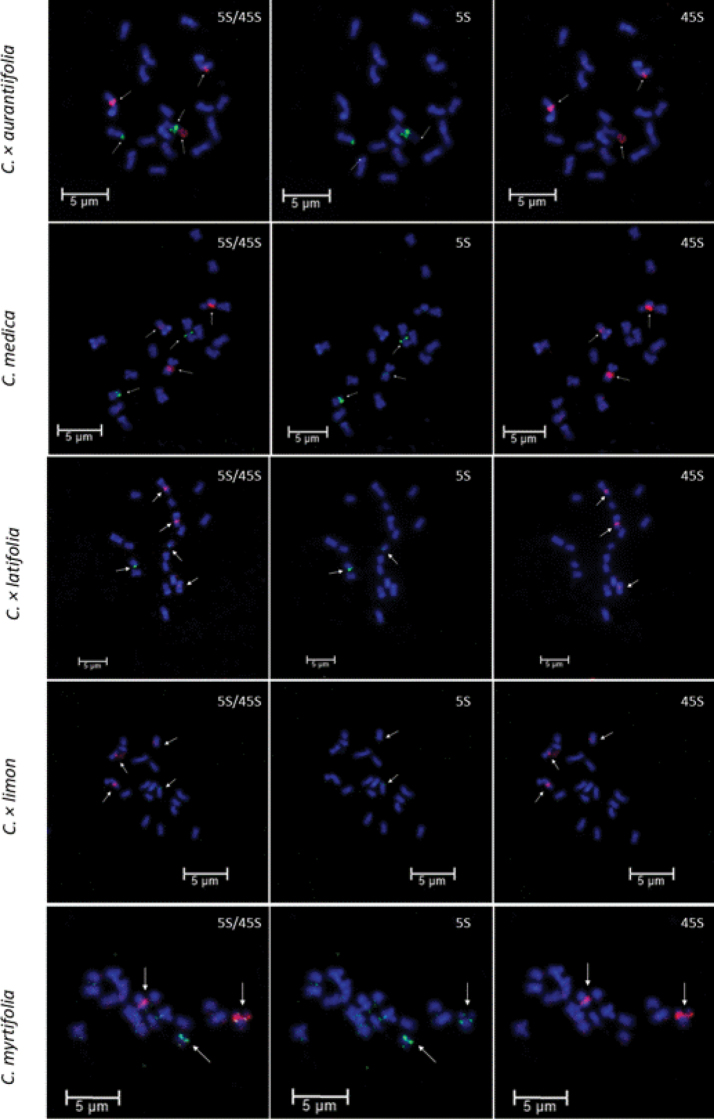
Chromosomal localization of 45S and 5S rDNAs in *C. × aurantiifolia*, *C. medica*, *C. × latifolia*, *C. × limon* and *C. myrtifolia*. FISH mapping using 45S rDNA (red) and 5S rDNA (green) on metaphase chromosomes. The white arrows indicate the 45S and 5S rDNA sites. Scale bar: 5 µm.

45S rDNA loci were located in the pericentromeric regions of chromosome pair 1 and chromosome 4 in *C. × aurantiifolia*, on chromosome pair 1 and chromosome 3 in *C. medica*, on chromosome pair 1 and on chromosomes 2 in *C. × latifolia*, on chromosomes 1 and 3 in *C. × limon*, and on chromosome pair 7 in *C. myrtifolia* (Fig. 2, Suppl. material 2: figs S6–S10).

**Figure 3.**
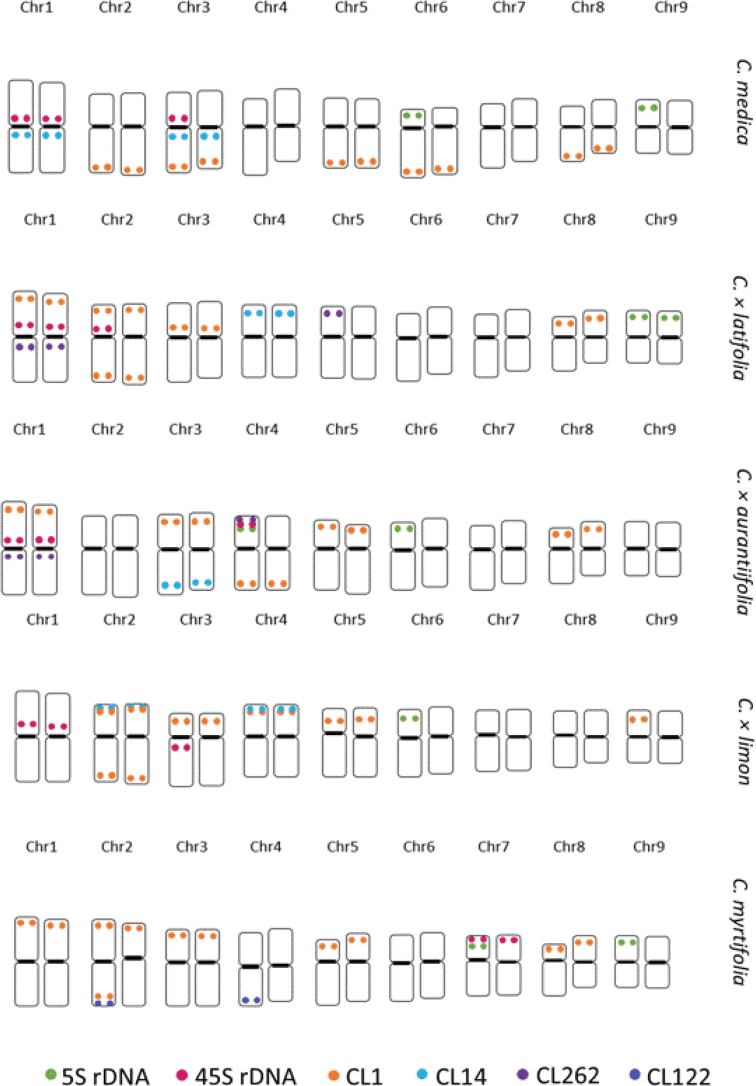
Ideograms with FISH signal tags in *C. × aurantiifolia*, *C. medica*, *C. × latifolia*, *C. × limon* and *C. myrtifolia*.

We developed a schematic karyotype ideogram for the investigated species (Fig. 3, Suppl. material 2: figs S1–S5).

## Discussion

### Cytogenetic markers in *Citrus*: tandem repeats and rDNA

In this study, several previously uncharacterized tandem repeats (CL1, CL14, CL262, and CL122) were identified and mapped on the chromosomes of five *Citrus* species using FISH. These repeats exhibited species-specific distribution patterns and, together with 45S and 5S rDNA markers, improved chromosome identification.

The identification of new tandem repeats expands the set of available chromosomal markers for *Citrus*, where cytogenetic analysis is complicated by small chromosome size and limited morphological differentiation. The chromosomal localization of identified tandem repeats relative to the 45S and 5S rDNA loci provides a more detailed cytogenetic framework. The combined use of these cytogenetic markers allows for more accurate identification of homologous chromosome pairs and facilitates comparative analysis across species.

### The relative positions of tandem repeats and rDNA loci on chromosomes

Hybridization is often accompanied by “genomic shock”, manifested as polymorphism in the arrangement of rDNA and tandem repeats. For example, variations in rDNA copy number have been described in response to selective pressure or stress (Deng et al. 2019; Wang et al. 2023). However, direct evidence of such processes in citrus remains limited. Our results demonstrate both the conservatism and modification of rDNA loci and specific repeats in closely related citrus taxa.

A comparison of the localization of tandem repeats with 45S and 5S rDNA regions revealed that different classes of repetitive DNA occupy distinct chromosomal domains. 45S rDNA loci were predominantly located in pericentromeric regions, while tandem repeats, particularly CL1 and CL122, were more frequently localized in subtelomeric regions.

Colocalization was more frequently observed between different tandem repeats, such as CL1 with CL14 and CL122, indicating the clustering of satellite DNA. At the same time, the CL262 repeat was detected in regions corresponding to 45S rDNA sites in lime species, although no sequence homology was identified, suggesting independent accumulation in structurally similar chromosomal regions.

The distribution patterns of the identified repeats revealed both conserved and species-specific features. The CL1 repeat was detected in all studied species and predominantly localized in subtelomeric regions, although its copy number varied between taxa. Such variability is characteristic of rapidly evolving satellite DNA, which can change in copy number and chromosomal position over relatively short evolutionary timescales (Lim et al. 2013; Biscotti et al. 2015).

In contrast, other repeats exhibited more restricted distributions. For example, CL14 showed different chromosomal localization patterns between *C. medica* and its hybrids, while CL262 was detected only in lime species, supporting their close relationship. The presence of species-specific repeats such as CL122 further reflects genomic divergence within the genus. In the *Citrus* species studied, the 5S and 45S rDNA loci were located on different chromosomes, although conservative linkage between them was observed in some members of the Aurantioideae Eaton, 1836 subfamily (Barros e Silva et al. 2013). This variation reflects the structural diversity within this group.

### rDNA organization in *Citrus* species and comparison with previous studies

*C. medica* is one of the four ancestral species of cultivated citrus and is considered a distinct biological species (Carvalho et al. 2005; Curk et al. 2016; Ollitrault et al. 2020). The rDNA distribution observed in *C. medica* is consistent with previous data indicating the absence of 5S–45S linkage (Carvalho et al. 2005; Deng et al. 2019). In contrast, hybrid species such as *C. × limon* and *C. × aurantiifolia* exhibit varying numbers and arrangements of rDNA loci, reflecting their complex genomic origins and structural variability (Carvalho et al. 2005; Curk et al. 2016; Ollitrault et al. 2020; Song et al. 2023).

Previous studies of *C. myrtifolia* identified two 5S rDNA regions located on different types of chromosomes and three 45S rDNA regions in the varieties studied (Moraes et al. 2007; Guerra et al. 2020). In our study of this species, two 5S and two 45S rDNA sites were detected.

*C. × latifolia* is a hybrid species derived from four ancestral taxa: *C. reticulata*, *C. maxima*, *C. medica*, and *C. micrantha*, comprising cultivars with both diploid and triploid genotypes (Ollitrault et al. 2020). Our FISH analysis revealed three 45S rDNA loci and one 5S rDNA locus in this species, with no observed co-localization.

The hybrid forms *C. × limon*, *C. × aurantiifolia*, and *C. × latifolia* have the number of 5S and 45S loci in the studied forms falls within the range previously documented for other *Citrus* taxa (Deng et al. 2019; Xiao et al. 2025). The relatively stable pattern of 45S rDNA compared to the more variable 5S loci represent a potentially useful characteristic for cytogenetic analysis.

## Conclusion

We identified and mapped new cytogenetic markers for the genus *Citrus*, including CL1, CL14, CL262, and CL122. In addition, we mapped the classical 5S and 45S rDNA markers in five species: *C. medica*, *C. × aurantiifolia*, *C. × limon*, *C. myrtifolia*, and *C. × latifolia*. Analysis of the distribution of rDNA and tandem repeats revealed conserved chromosomal localization of 45S and 5S rDNA loci, in contrast to the greater variability observed in the chromosomal distribution of tandem repeats. The observed differences in repeat distribution among species, particularly in hybrid taxa, are consistent with genome restructuring processes associated with hybridization and the dynamic evolution of repetitive DNA.

The obtained FISH profiles demonstrate that the identified repeats represent useful cytogenetic markers for karyotype characterization and comparative analysis in *Citrus*.

## Additional information

### Artificial Intelligence (AI) use

The authors accept full responsibility for the content of the manuscript, including the disclosure of any use of AI.

Regarding the use of AI in the preparation of this manuscript, the authors declare the following:

We used the DeepSeek AI model (DeepSeek R1) to ensure linguistic accuracy and compliance with the academic style of the article. The model was asked to translate the original Russian text using a specially designed set of instructions, paying special attention to the passive voice, technical accuracy and conciseness. The questions were as follows: 1. Criticize the revised statement as a reviewer, pointing out any shortcomings or areas requiring further improvement. *Initial draft* 2. Translate the following Russian text into formal, academic English of a scientific paper. Use active voice where appropriate, maintain precise scientific terminology, and ensure the text is concise and objective.

### Author ORCIDs

Y.N. Smotrova https://orcid.org/0009-0005-9179-2203

M.Gr. Monakhos https://orcid.org/0009-0003-6604-0822

O.V. Razumova https://orcid.org/0000-0001-5157-0087

D.V. Romanov https://orcid.org/0000-0002-7495-7572

### Data availability

All of the data that support the findings of this study are available in the main text or Supplementary Information.

## Supplementary materials

Supplementary material 1 Rutaceae plant collection located in All-Russia Research Institute of Agricultural Biotechnology. Data type xlsx https://binary.pensoft.net/file/1702999This dataset is made available under the Open Database License (http://opendatacommons.org/licenses/odbl/1.0/). The Open Database License (ODbL) is a license agreement intended to allow users to freely share, modify, and use this Dataset while maintaining this same freedom for others, provided that the original source and author(s) are credited. Yuliya N. Smotrova, Mikhail Gr. Monakhos, Olga V. Razumova, Dmytry V. Romanov

Supplementary material 2 Supplementary figures Data type pdf Explanation note **fig. S1**. Karyotype of *C. medica*. Scale bar = 5 μm. **fig. S2**. Karyotype of *C. × limon*. Scale bar = 5 μm. **fig. S3**. Karyotype of *C. × aurantiifolia*. Scale bar = 5 μm. **fig. S4**. Karyotype of *C. × latifolia*. Scale bar = 5 μm. **fig. S5**. Karyotype of *C. myrtifolia*. Scale bar = 5 μm. **fig. S6**. An example of *C. medica* metaphase plates. Scale bar = 5 μm. **fig. S7**. An example of *C. × limon* metaphase plates. Scale bar = 5 μm. **fig. S8**. An example of *C. × aurantiifolia* metaphase plates. Scale bar = 5 μm. **fig. S9**. An example of *C. × latifolia* metaphase plates. Scale bar = 5 μm. **fig. S10**. An example of *C. myrtifolia* metaphase plates. Scale bar = 5 μm. https://binary.pensoft.net/file/1703000This dataset is made available under the Open Database License (http://opendatacommons.org/licenses/odbl/1.0/). The Open Database License (ODbL) is a license agreement intended to allow users to freely share, modify, and use this Dataset while maintaining this same freedom for others, provided that the original source and author(s) are credited. Yuliya N. Smotrova, Mikhail Gr. Monakhos, Olga V. Razumova, Dmytry V. Romanov
