# Supplementary material for: Fish mapping of rdna and novel tandem repeats in Citrus species
Source: Comp Cytogenet. 2026 Jun 30;20:157–70. doi: 10.3897/compcytogen.20.190408 (PMC13342924; doi:10.3897/compcytogen.20.190408)
Supplement: Supplementary material 2 — Supplementary figures [file comparative_cytogenetics-20-157_article-190408__-s002.pdf]

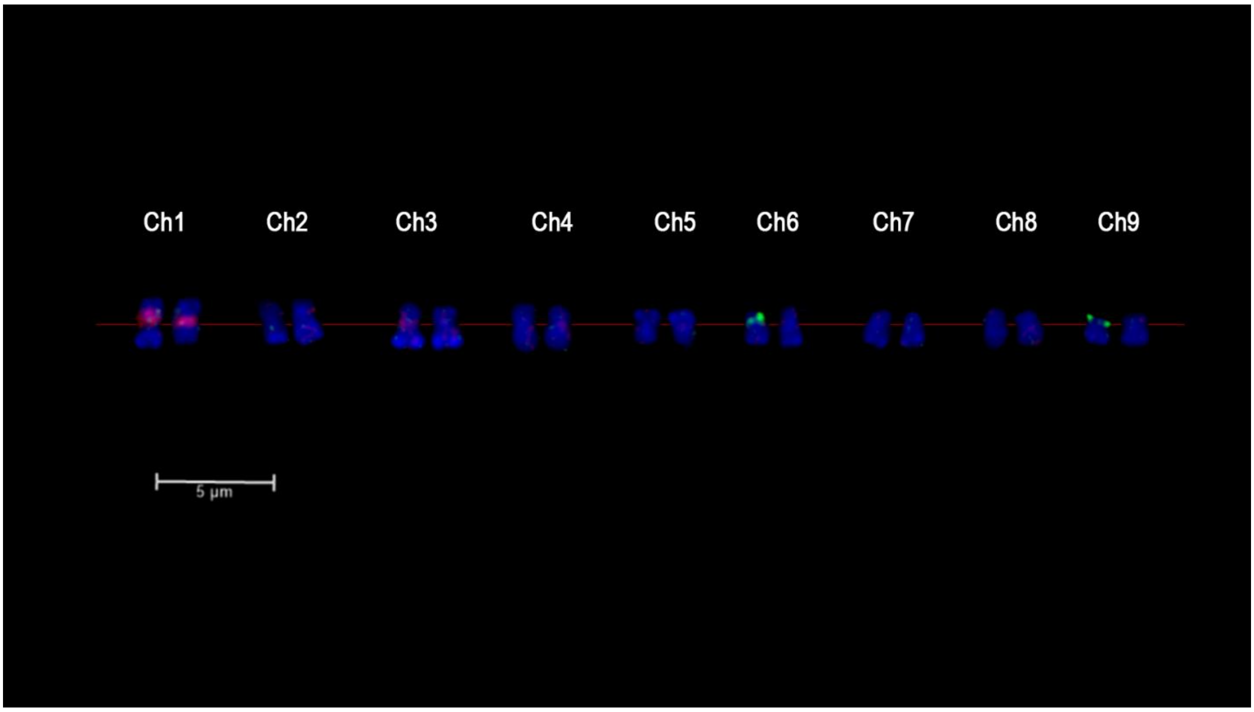

Figure 1S. Karyotype of *C. medica*. Scale bar = 5 µm.

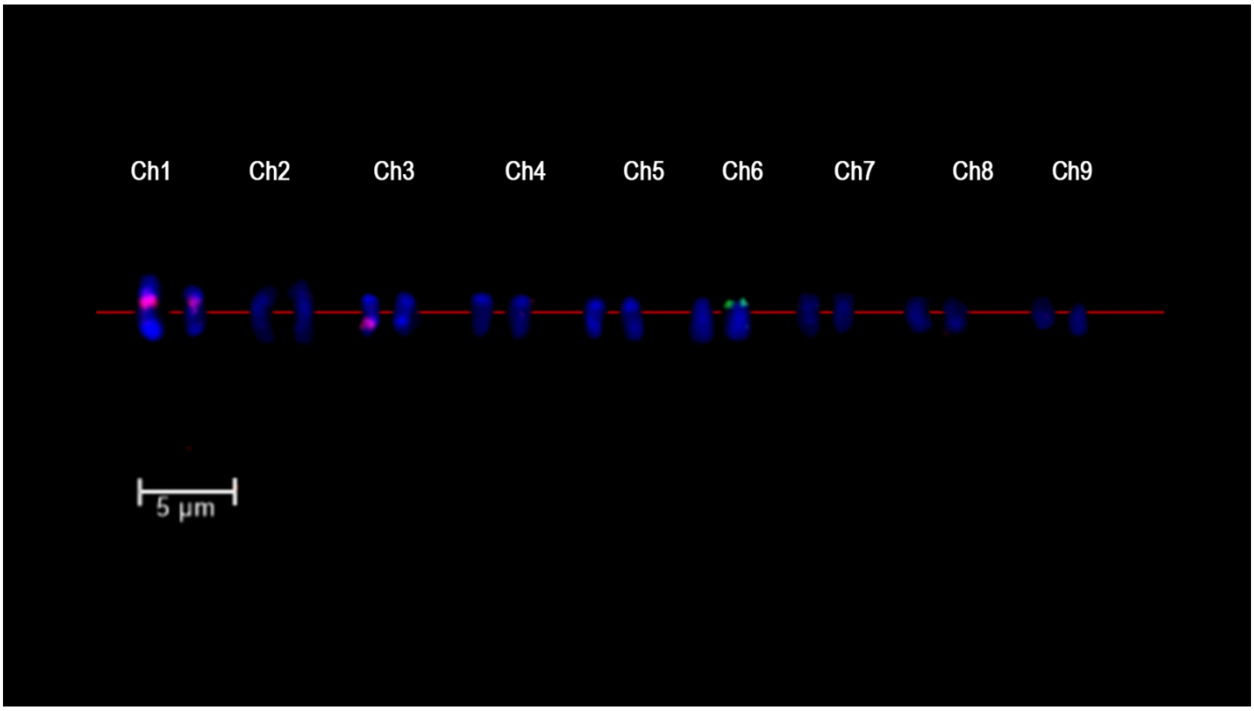

Figure 2S. Karyotype of *C. × limon*. Scale bar = 5 µm.

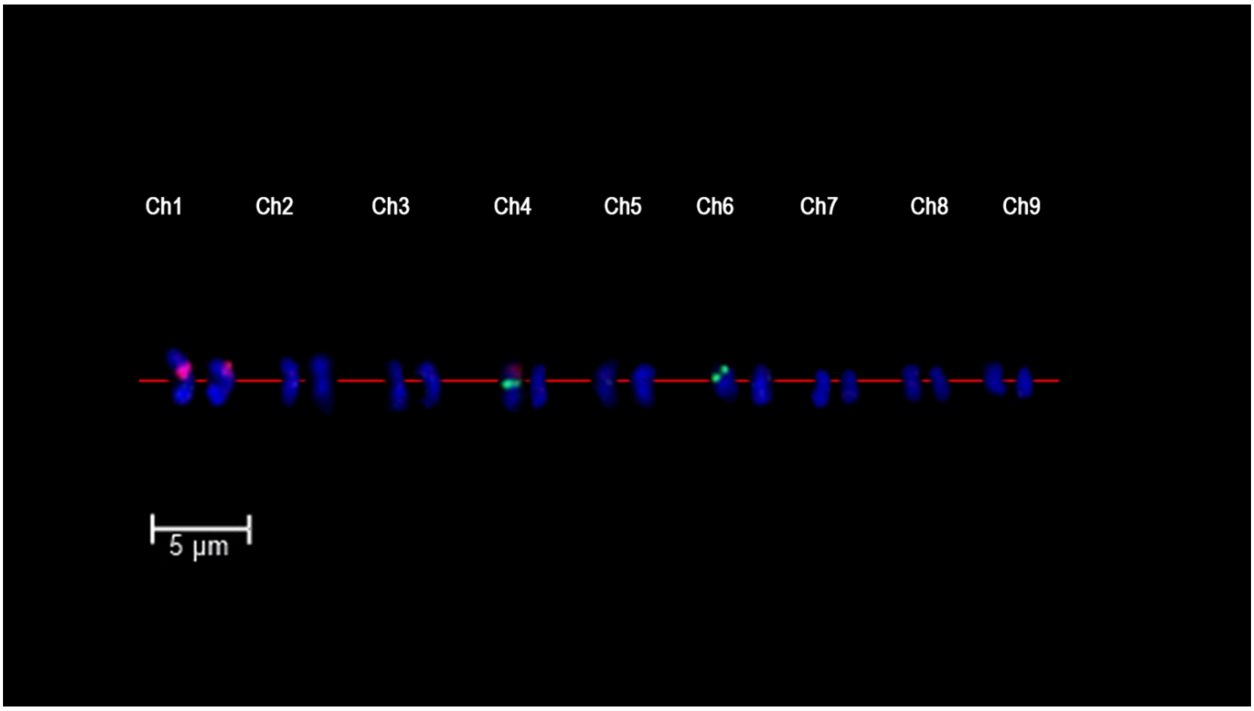

Figure 3S. Karyotype of *C.* × *aurantiifolia*. Scale bar = 5 µm.

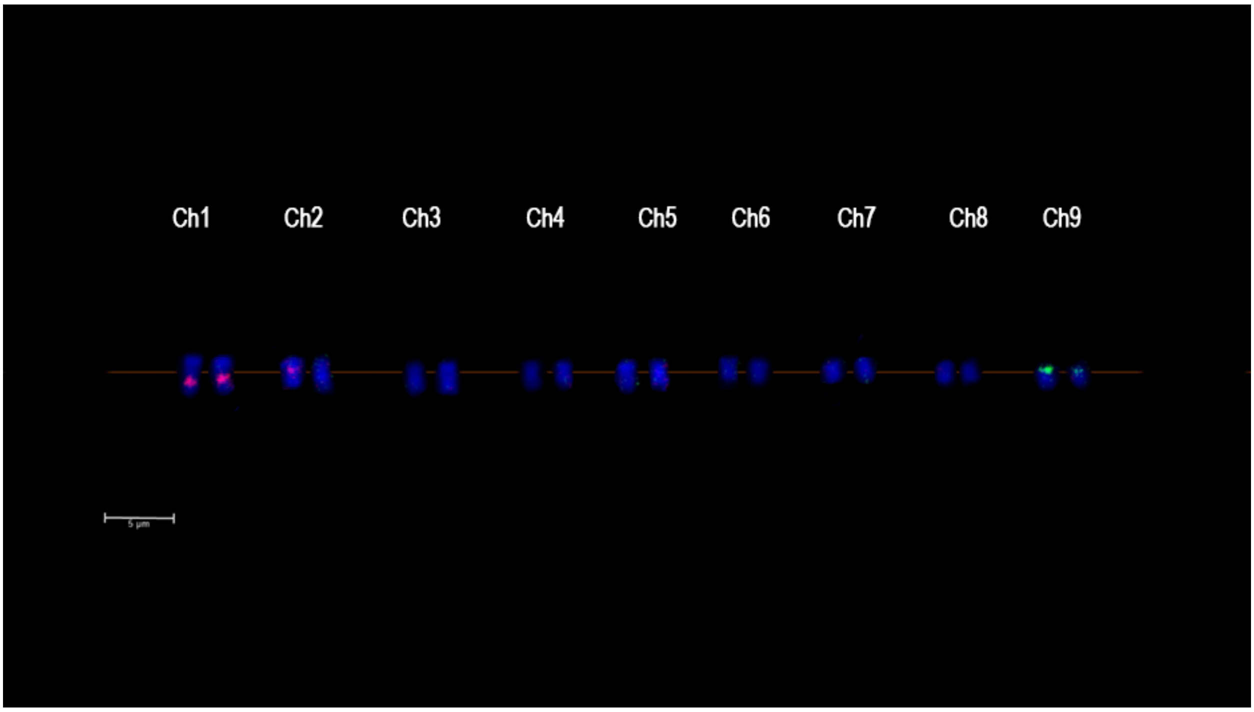

Figure 4S. Karyotype of *C.* × *latifolia*. Scale bar = 5 µm.

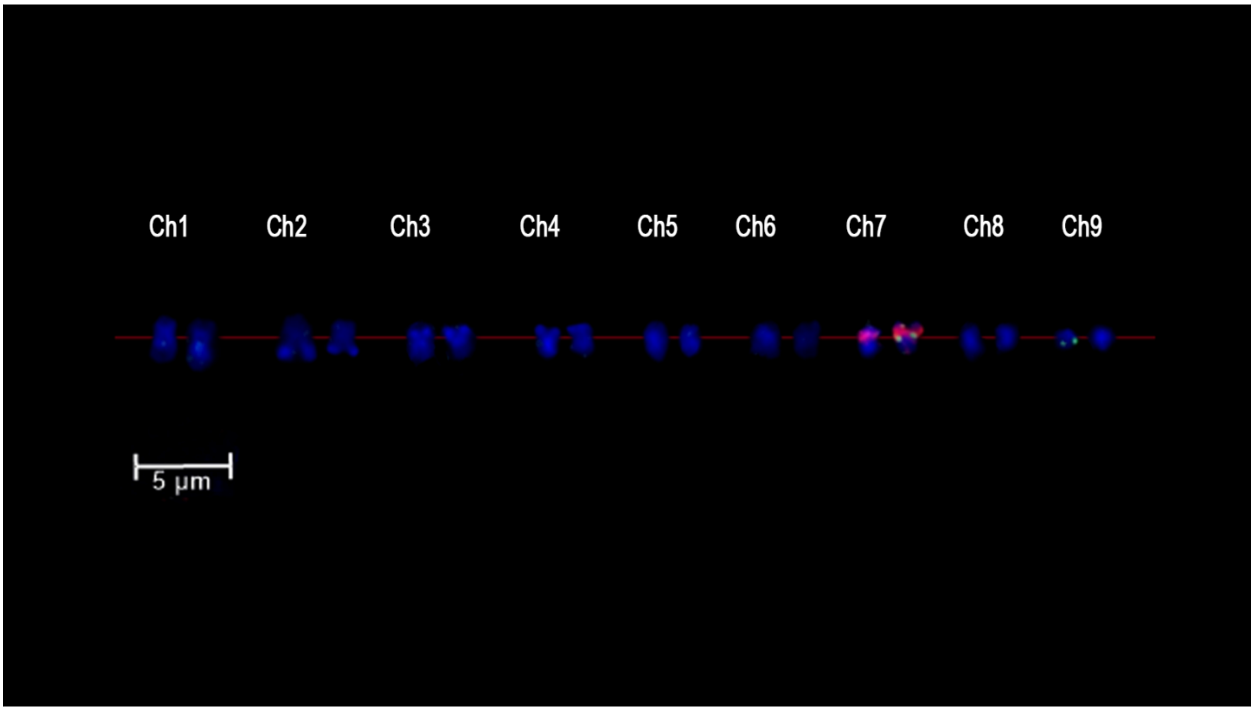

Figure 5S. Karyotype of *C. myrtifolia*. Scale bar = 5 µm.

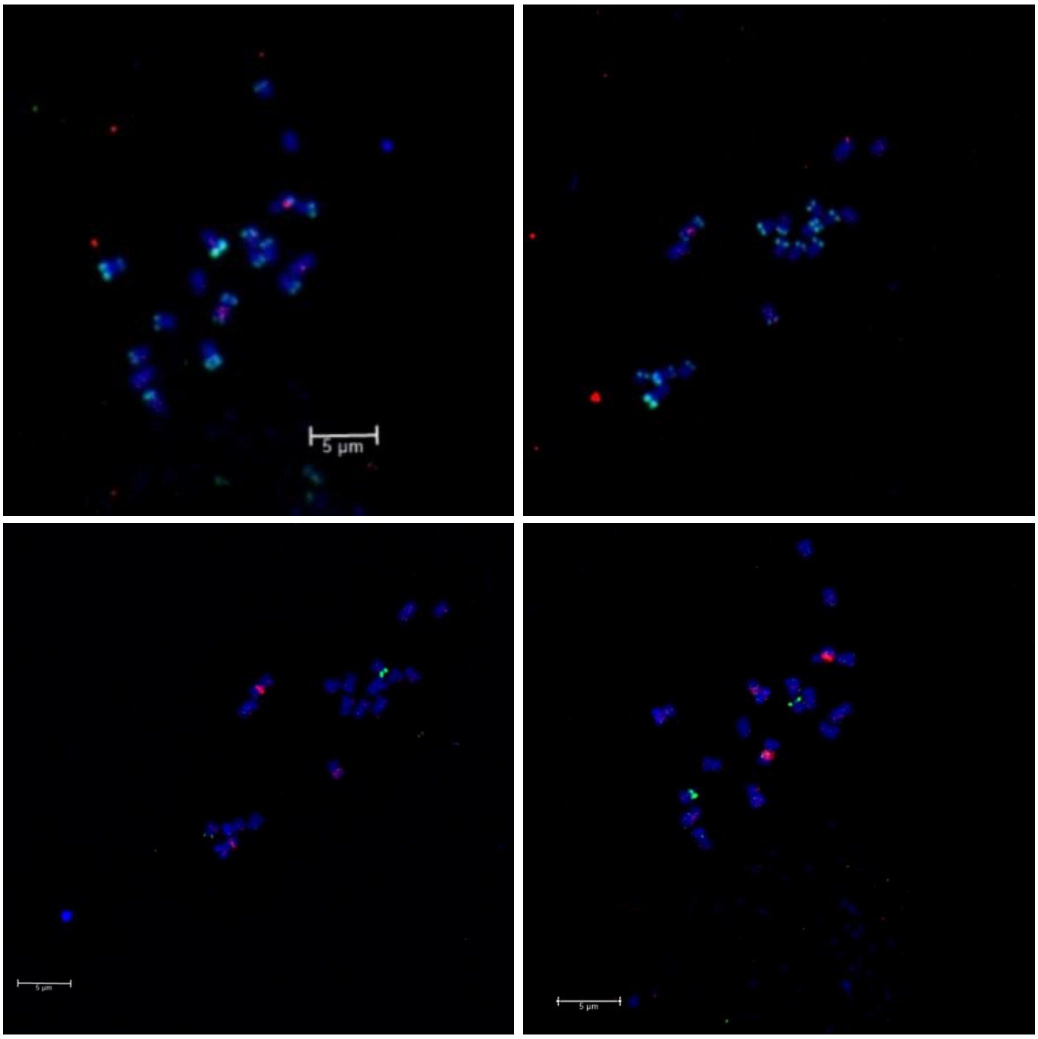

Figure 6S. An example of *C. medica* metaphase plates. Scale bar = 5 µm.

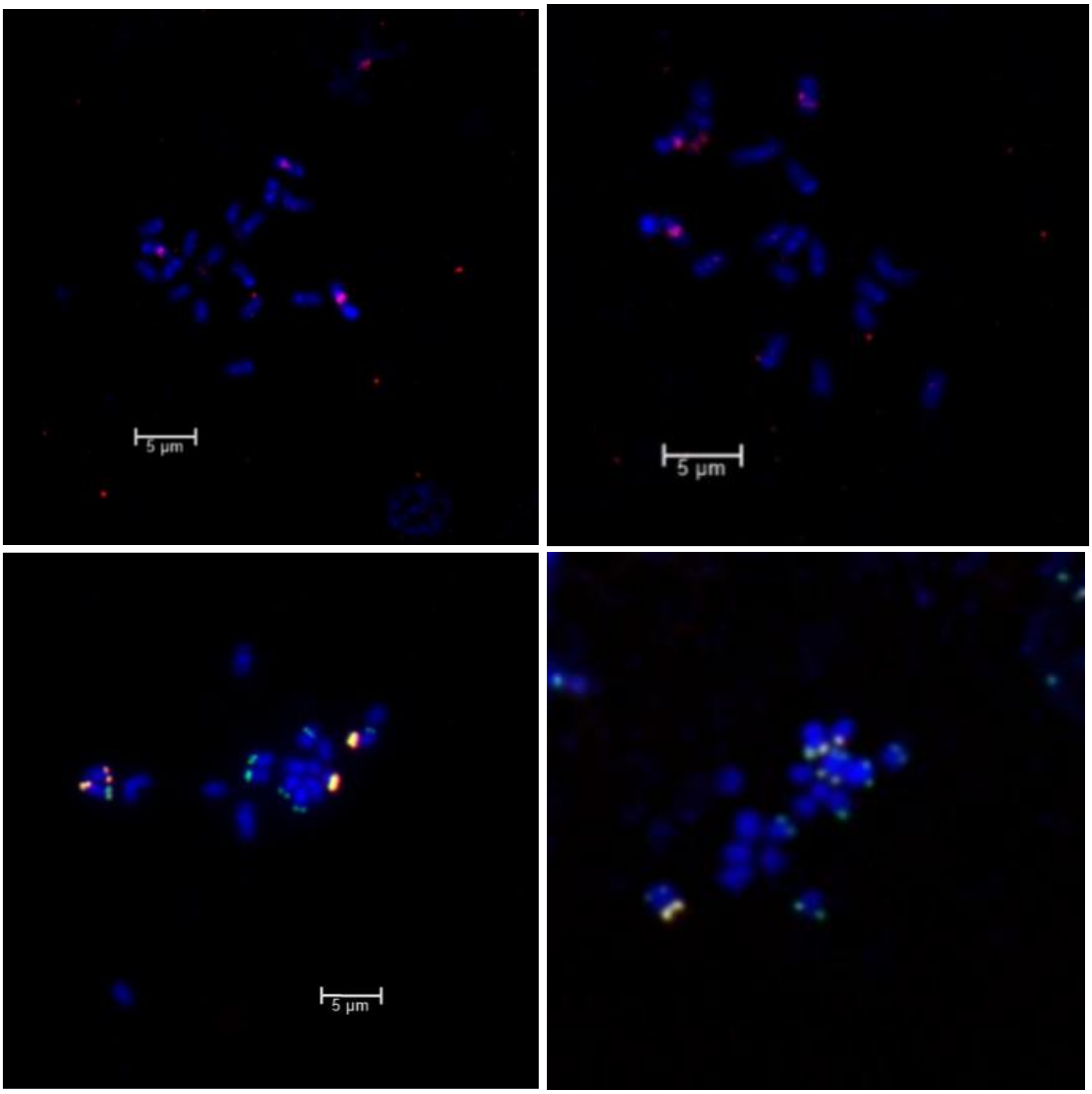

Figure 7S. An example of *C.* × *limon* metaphase plates. Scale bar = 5 µm.

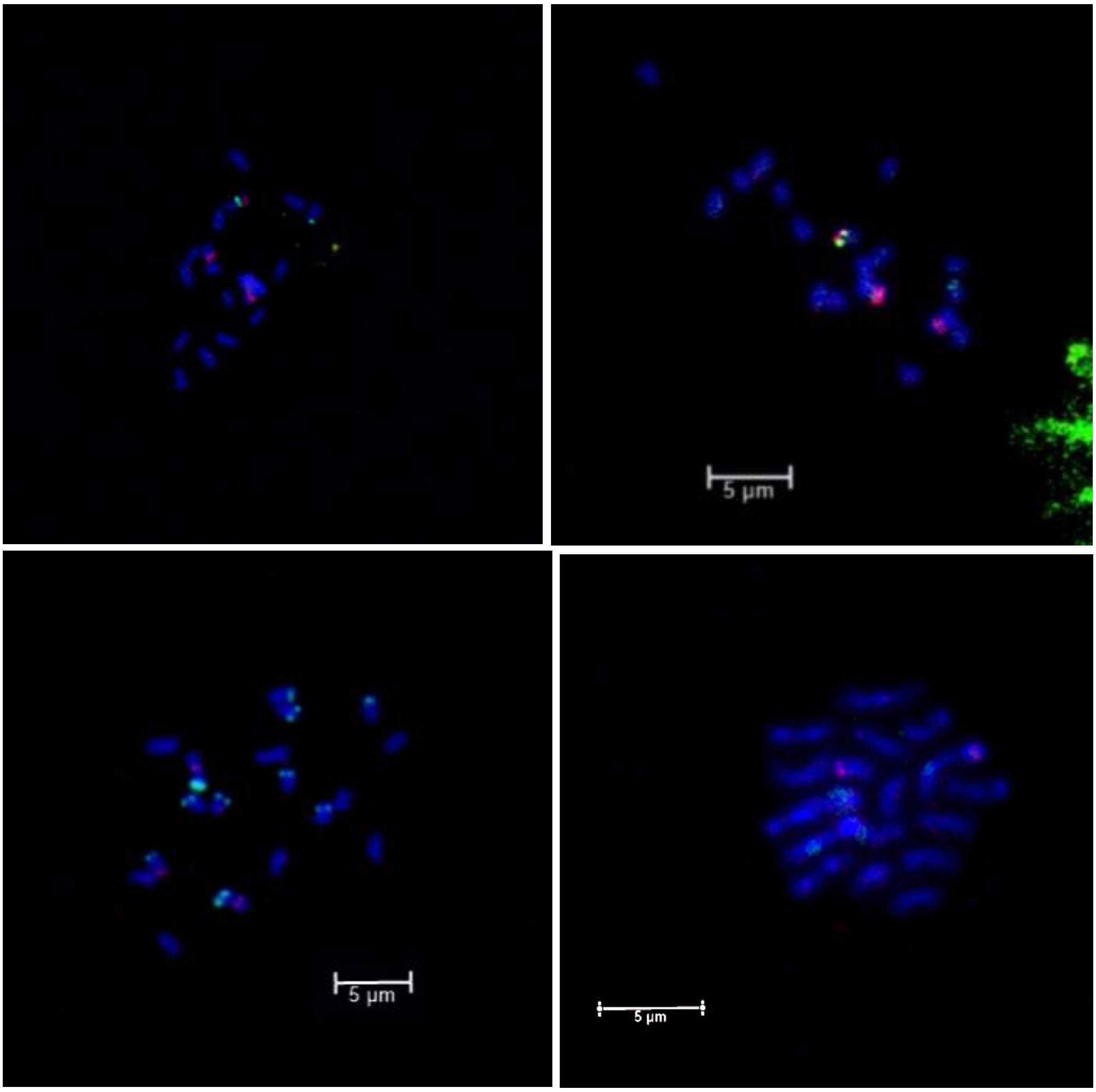

Figure 8S. An example of *C.* × *aurantiifolia* metaphase plates. Scale bar = 5 μm.

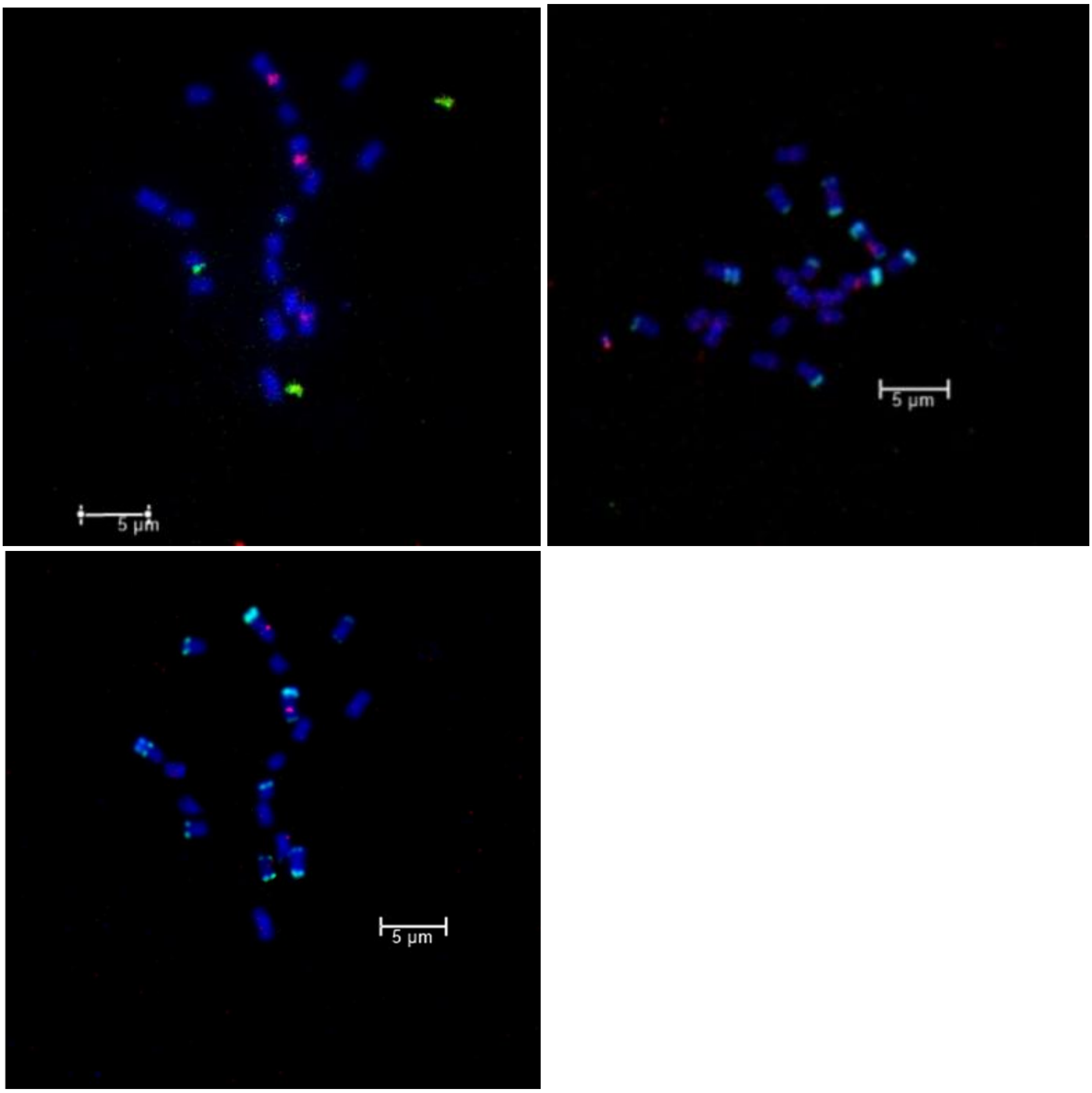

Figure 9S. An example of *C.* × *latifolia* metaphase plates. Scale bar = 5 µm.

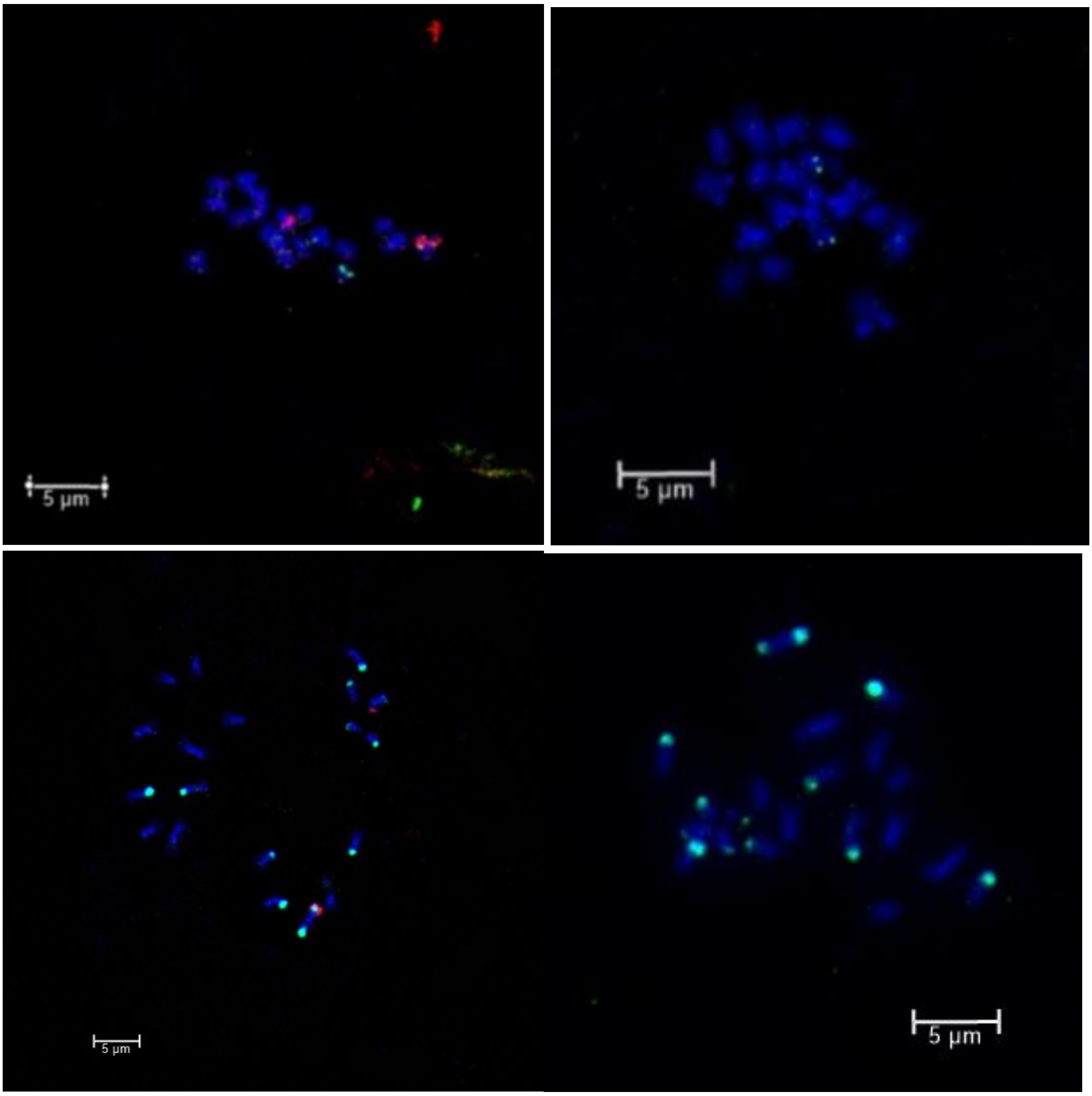

Figure 10S. An example of *C. myrtifolia* metaphase plates. Scale bar = 5 µm.
